# Perfluorocarbon nanodroplets are cytocompatible with osteoblast-lineage cells and modulate *in vitro* osteoclastogenesis differentially in normoxia and hypoxia

**DOI:** 10.1038/s41598-026-57136-7

**Published:** 2026-06-15

**Authors:** Kirsten O’Brien, Helen J Knowles, Sam Sloan, Robin M H Rumney, Dario Carugo, Eleanor Stride, Nicholas D Evans

**Affiliations:** 1 Bone and Joint Research Group, Faculty of Medicine, University of Southampton, Southampton, UK; 2 School of Engineering, University of Southampton, Southampton, UK; 3 Nuffield Department of Orthopaedics, Rheumatology and Musculoskeletal Sciences (NDORMS), University of Oxford, Oxford, UK; 4 Institute of Biomedical Engineering, University of Oxford, Oxford, UK; 5 Institute for Life Sciences, University of Southampton, Southampton, United Kingdom

**Keywords:** nanodroplets, oxygen-delivery, hypoxia, drug-delivery, bone, fracture healing, Cell biology, Medical research, Stem cells

## Abstract

Perfluorocarbon nanodroplets (PFC NDs) are submicrometre particles comprising a liquid perfluorocarbon core stabilised by a phospholipid shell, with emerging potential for therapeutic targeted gas delivery and drug delivery. While related PFC microbubbles have demonstrated promise in drug delivery applications, including bone repair, the biological effects and translational relevance of PFC nanodroplets in skeletal systems remain untested. This study investigated the effects of nanodroplets on bone cell viability in vitro and on osteoclastogenesis under normoxic and hypoxic conditions. Confocal microscopy and flow cytometry were used to assess nanodroplet association with skeletally-relevant MC3T3-E1 and Saos-2 osteoblastic cell lines, human bone-marrow-derived stromal cells, and peripheral blood mononuclear cell-derived osteoclasts. Cellular viability and differentiation were evaluated using Alamar Blue, TRAP, and DAPI staining. Long-term (12-day) nanodroplet exposure significantly reduced osteoclast number in both normoxia and hypoxia, whereas short-term exposure in hypoxia increased osteoclast formation. Importantly, nanodroplets did not adversely affect osteoblastic viability. In summary, these findings indicate that PFC nanodroplets are compatible with key skeletal cell populations and can modulate osteoclastogenesis in a context-dependent manner, supporting their potential as vehicles for bone-targeted gas or drug delivery.

## Introduction

 Bone fractures resulting in delayed or non-union healing present a significant socioeconomic burden. Between 2004 and 2014, over 2.4 million people presented with a bone fracture in England^1^. While most bone fractures heal normally, 2–10% (depending on site and disease context) result in non-union^2–5^. Not only is treatment expensive, with costs per patient estimated at between £15,566 to £17,200 for humeral and femoral fractures respectively in the UK^6^, but patients also endure chronic pain and have difficulties going about daily activities^4,7^. Despite this, there is currently no proven systemic therapy available for treatment of bone repair.

Perfluorocarbon nanodroplets have been the subject of growing research interest for drug delivery in recent years, with a focus on their use as cavitation nuclei, drug delivery agents and oxygen carriers^8–11^. Nanodroplets typically consist of submicrometre particles with a liquid perfluorocarbon core, stabilised by a phospholipid shell. When exposed to ultrasound, nanodroplets undergo vaporisation to form microbubbles that cavitate under continued ultrasound stimulation (often referred to as phase-change nanodroplets). They have the advantage of longer circulation half-lives than microbubbles, they are able to permeate deeper into tissue, and are less likely to be detected by the immune system^12–14^. Moreover, they can be loaded with drugs that can be released upon ultrasound activation allowing for precise control over the time and location of drug delivery.

To date, nanodroplet-based therapies have been studied primarily in oncology. For example, Huang et al. found that hydralazine-loaded nanodroplets significantly inhibited tumour growth in both cultured cancer cells and tumour-bearing mice^15^. In vitro studies using antibody-conjugated phase-change nanodroplets have shown selective internalisation into high-antigen-expressing cancer cells and, following ultrasound-induced vaporisation, substantial reductions in cell viability (e.g. 57% cell toxicity in high-expressing lines) compared with non-activated controls^16^. Nanodroplets accumulate in tumours partly through the enhanced permeation and retention (EPR) effect, whereby fast-growing and poorly organised vasculature exhibits increased permeability^17,18^, as well as a result of macrophage-mediated retention^19^. As these effects are also evident in bone repair^20^, nanodroplets may also be suitable for targeted delivery to heal bone.

Another potentially attractive mechanism by which nanodroplets may support effective drug delivery is related to their oxygen-carrying capacity. Perfluorocarbons dissolve large quantities of oxygen, approximately 50 times more than water and 2.5 times more than blood^21,22^. For this reason, nanodroplet-dependent oxygen delivery has been studied in vivo for the treatment of tumour hypoxia, which is often a limiting factor for therapeutic efficacy. Huang et al. investigated oxygen delivery using nanodroplets containing doxorubicin (DOX) hydrochloride and perfluorotributylamine (PFTBA) for chemosonodynamic therapy, and observed significantly reduced HIF-1α expression compared with treatment using DOX-only nanodroplets^23^. Despite substantial progress in oncological applications, little is known about the interactions between nanodroplets and bone or bone cells.

This is pertinent because local oxygen tension is a critical regulator of skeletal repair, influencing angiogenesis, osteogenic differentiation, and coupling between vascular and bone formation. Experimental stabilisation of hypoxia-inducible factor (HIF) signalling enhances vascularisation and bone regeneration in vivo, demonstrating that modulation of oxygen availability can directly promote fracture healing^24^. Conversely, persistent hypoxia or impaired revascularisation is strongly associated with delayed union and non-union, highlighting oxygen delivery as a key therapeutic target in regenerative orthopaedics^25^. These observations suggest that biomaterial or carrier systems capable of locally transporting oxygen may provide a mechanistically grounded strategy to enhance bone repair. Perfluorocarbon nanodroplets represent a plausible platform for skeletal drug and gas delivery, motivating investigation of their direct interactions with bone-relevant cells and regenerative processes.

In this study, we investigated the potential of perfluoropentane nanodroplets as therapeutic delivery agents for bone repair. We tested the hypothesis that nanodroplets are compatible with key skeletal cell populations and can modulate osteoclastogenesis without impairing osteoblastic viability, supporting their potential as carriers for targeted skeletal drug or gas delivery.

## Results

**Fig. 1:**
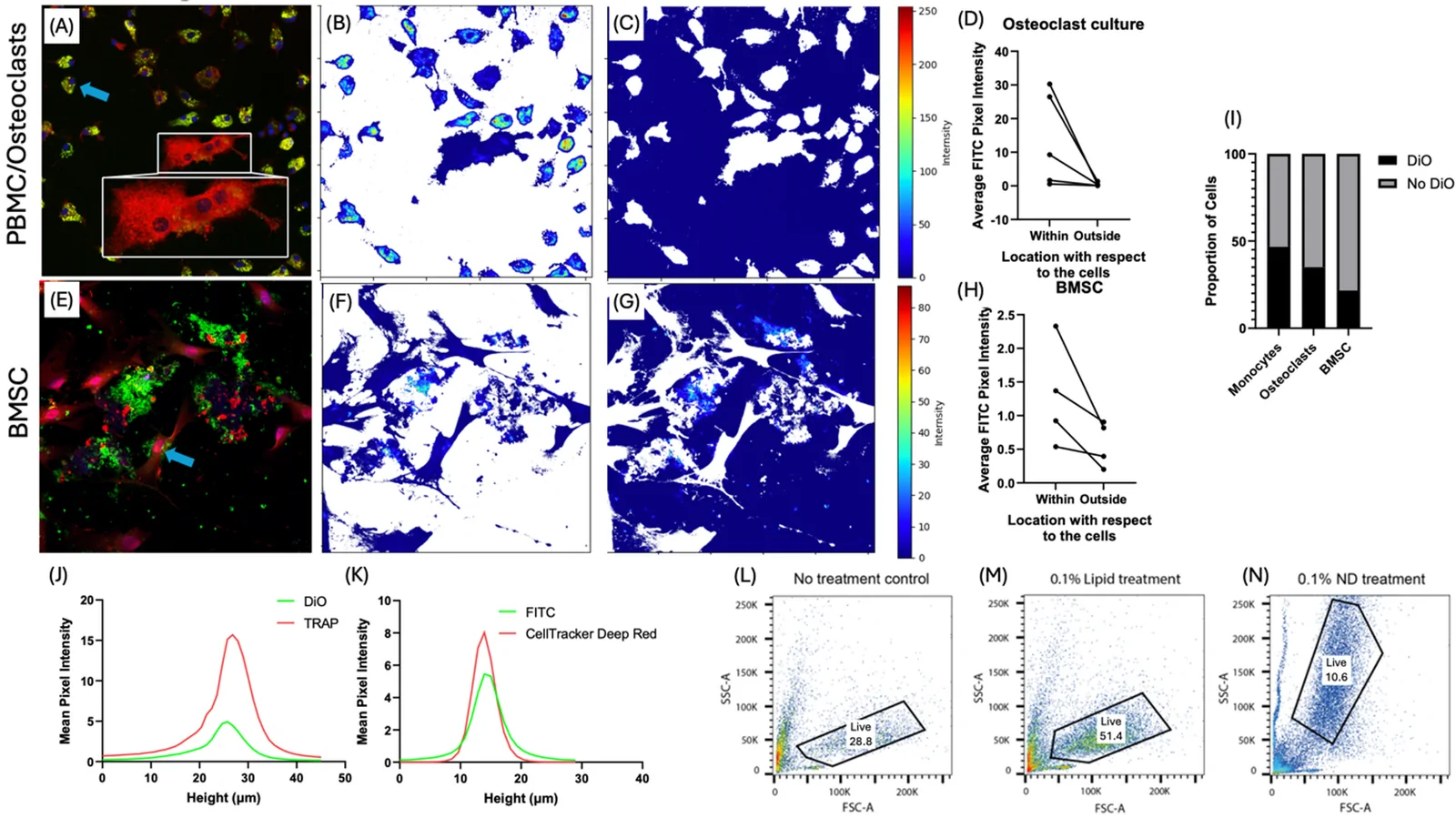
Nanodroplets weakly associate with bone marrow stromal cells and osteoclasts but strongly associate with monocytes. Maximum projection confocal images of (A) PBMC-derived osteoclast culture with a monocyte highlighted by a blue arrow and an osteoclast highlighted to demonstrate slight DiO fluorescence and (E) bone marrow stromal cells. The cell specific stain (TRAP for osteoclasts and cell tracker deep red for BMSCs) was used to create a region of interest and the FITC intensity (B, F) within and (C, G) outside of the red region of interest is visualised. The (D, H) average DiO intensity was quantified (Osteoclast culture: *p* = 0.0954; BMSC: *p* = 0.1442). (I) Proportion of DiO^+^ cells by cell type. (J) The DiO and TRAP and (K) DiO and CellTracker Deep Red intensity in the Z direction. *N* = 5. (L-N) The side scatter vs. front scatter of monocytes treated with unlabelled nanodroplets/lipids. Biological repeats (N) = 3, Technical repeats (n) = 3.

To determine whether nanodroplets directly interact with cells, BMSCs and PBMC-derived osteoclast cultures were exposed to DiO-labelled nanodroplets for 72 h and imaged using a confocal microscope. PBMC-derived osteoclast cultures were exposed to fluorescent nanodroplets at day 9, resulting in a mix of monocytic and osteoclastic cells within the culture. DiO fluorescence coincided with the cells, with the highest intensity observed in the mononuclear cells by fluorescence microscopy (Fig. 1A). Using TRAP staining, a cellular region of interest was established (example images are shown in Fig. 1B, C). As shown in Fig. 1D, the DiO intensity was consistently higher within the TRAP^+^ cells than outside them (paired Wilcoxon signed-rank test, two-tailed, five independent experiments, *p* = 0.063; not statistically significant). Similarly, in BMSCs, although there was a trend for an increased DiO intensity within cell regions (Fig. 1E), this was not significant (Fig. 1H; *p* = 0.125). Quantification of DiO^+^ cells by cell type however (Fig. 1I) revealed a significantly higher affinity for the PBMC-derived monocytes (47% of monocytes) compared with PBMC-derived osteoclasts (35%) (*p* < 0.0001). It should be noted that the apparent reduction in signal intensity may partly reflect the greater spread of fluorescence over the larger area of osteoclasts. Finally, the weakest association was observed in the BMSC culture (22%).

In both the BMSC and osteoclast cultures, the red stain (CellTracker Deep Red for BMSC and TRAP for osteoclasts) was intracellular. Z-profile analysis (Fig. 1J, K) demonstrated that the DiO colocalised with the red stain in the z-direction, implying the DiO is intracellular and the nanodroplets are internalised by cells.

To test the hypothesis that macrophages associate with the nanodroplets, CD14^+^ PBMCs were cultured with MCSF only for 7 days to force a macrophage lineage. They were treated with nanodroplets (DiO-free) for the final 48 h before undergoing flow cytometric analysis. Side scatter increased with nanodroplet treatment compared to both the no treatment and lipid-only treatment controls (Fig. 1L-N), indicative of an increase in granularity as a result of the nanodroplets associating with the macrophages. Together, this data provides evidence of nanodroplet-cell association, particularly with monocyte-derived cells.

**Fig. 2:**
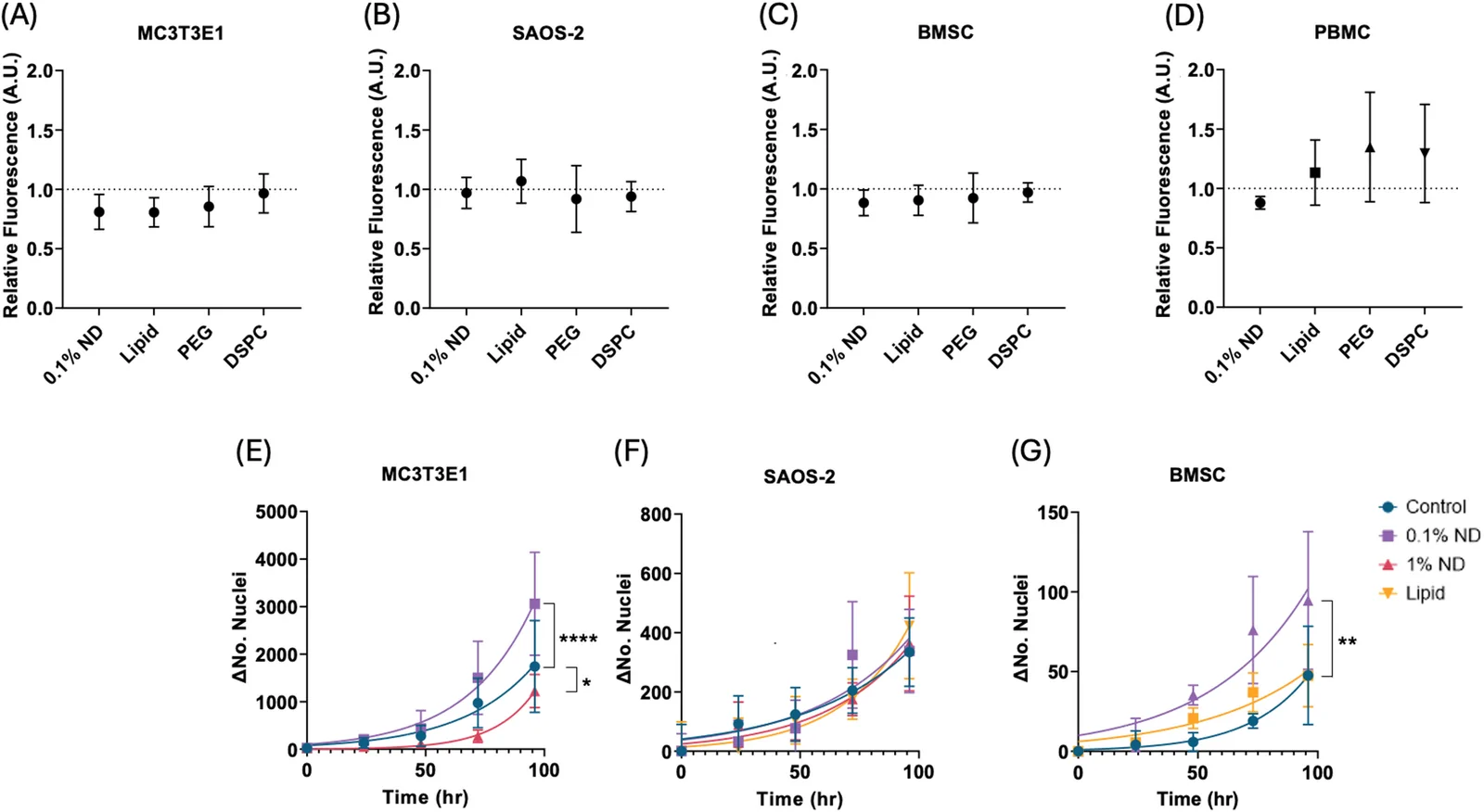
Effect on nanodroplets and their constituents on bone cell metabolism (A-D) and number (E-H). MC3T3E1 cells (A), Saos-2 cells (B), BMSCs (C), and PBMCs (D) were cultured with nanodroplets for 24 h in normoxia and assessed for metabolism using Alamar Blue. Dashed lines indicate the fluorescence value of the no-treatment control group to which the treatment groups are normalised. Cell number was quantified using DAPI staining for MC3T3E1, Saos-2 and BMSC cells treated for 4 days (E-G). Error bars represent one standard deviation from the mean (A-G). *p < 0.05, **p < 0.01 ***p < 0.001, ****p < 0.0001. *N* = 3, *n* = 6 for Alamar Blue; *N* = 3, *n* = 3 for DAPI.

Given that the nanodroplets associate with BMSCs, CD14^+^ PBMCs and osteoclasts, we next investigated whether this association impacted on cell viability. In previous work, no cytotoxic effect of nanodroplets was found on tumour or kidney cells^23,26^ but their effect on lineages of cells found in bone has not been reported to the best of our knowledge. To test the hypothesis that nanodroplets do not have an effect on the viability of bone cells, a panel of skeletally relevant cell types, including the. The murine osteoblast precursor cell line (MC3T3E1), the human osteosarcoma cell line (Saos-2), primary human bone marrow stromal cells (BMSC), and primary peripheral blood mononuclear cells (CD14^+^ PBMC) were incubated with nanodroplets and their metabolism was measured using Alamar Blue. Cell metabolism was not significantly affected by nanodroplets or their individual components, DSPC or PEG(40)s, in all cell types (Fig. 2A-D). In parallel, cell number was measured by counting DAPI-stained nuclei in MC3T3E1 and Saos-2 cells. MC3T3E1 proliferation increased on incubation with 0.1% (v/v) nanodroplets (1.76-fold increase, *p* < 0.0001) but was inhibited at a concentration of 1% (v/v) (1.42-fold decrease, *p* = 0.0227) compared to the no-treatment control (Fig. 2E). This effect was not observed in Saos-2 cells (Fig. 2F). In BMSCs, an increase in proliferation with nanodroplet treatment was observed with a 1.93 factor increase in the average number of nuclei (Fig. 2G: **p = 0.0014 at 96 h). Overall, these data support the hypothesis that nanodroplets have little effect on short-term cell viability but have significant effects on longer term proliferation of osteoblastic cells.

**Fig. 3:**
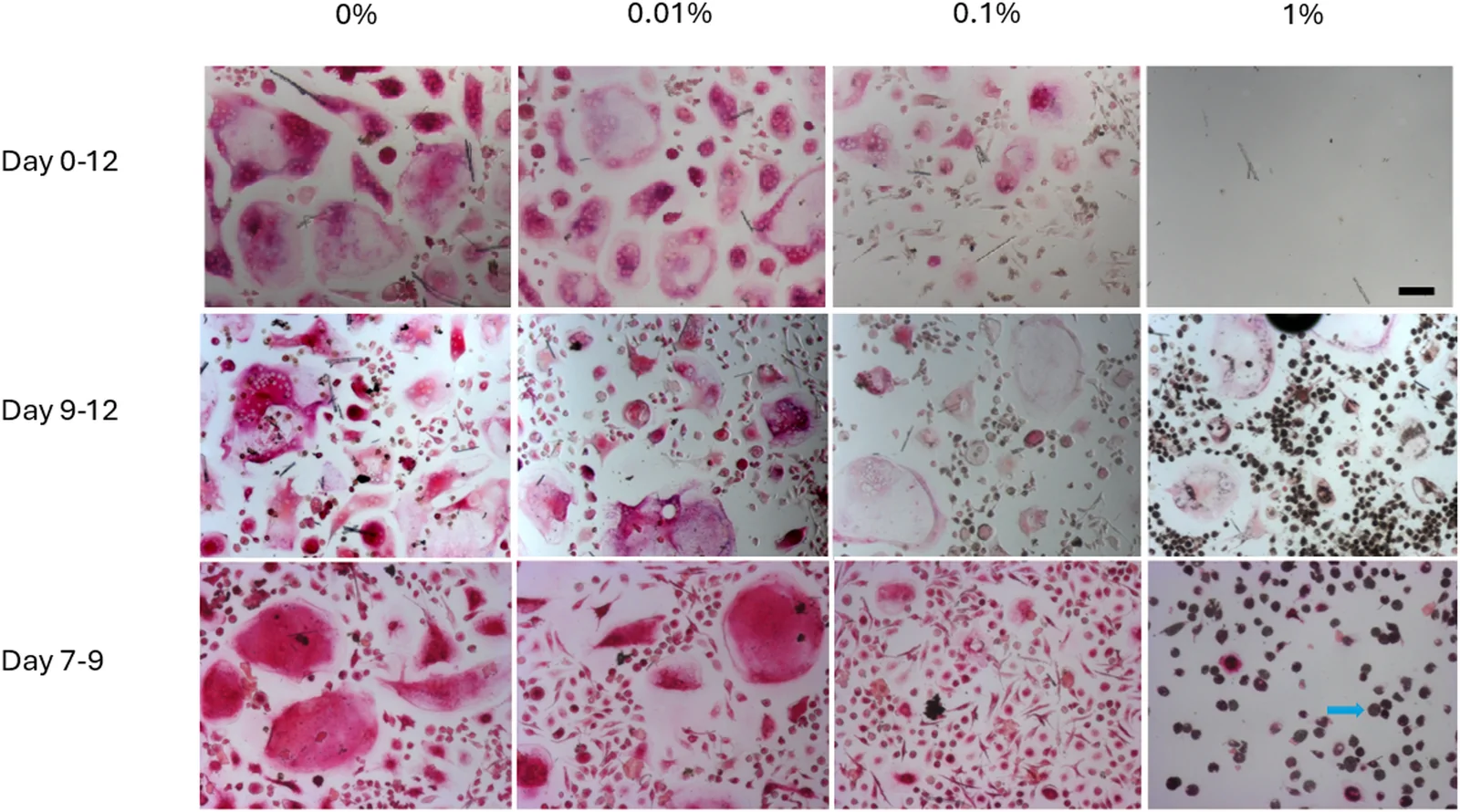
Nanodroplets inhibit fusion of osteoclasts in low nanodroplet concentrations, and suggest cytotoxicity at 1%. Images from 12-day osteoclast culture treated with nanodroplets for the full 12-day treatment (0–12), treating mature osteoclasts only (9–12) and intermittent treatment on fusing osteoclasts (7–9). The blue arrow indicates an example of nanodroplet-cell association evidenced by increased opacity, likely caused by nanodroplet accumulation in the mononuclear cells. Scale bar: 50 μm.

Although we observed association of nanodroplets with CD14^ +^ PBMCs and their osteoclast derivatives, no effect on cell metabolism was observed (Fig. 2D). We therefore hypothesised that nanodroplets would have no effect on osteoclast number. In direct contrast, however, by microscopic examination, we observed that nanodroplets caused an overt reduction in the number of osteoclasts, especially for the treatment regimens that were applied during osteoclast fusion (day 0–12 and day 7–9) where there was a clear reduction in the number of large multinuclear TRAP-positive cells in the culture (Fig. 3). Nanodroplet accumulation in the monocytes can also clearly be seen especially in the 1% (v/v) treatment group with the nanodroplets causing the cells to be dark in appearance.

**Fig. 4:**
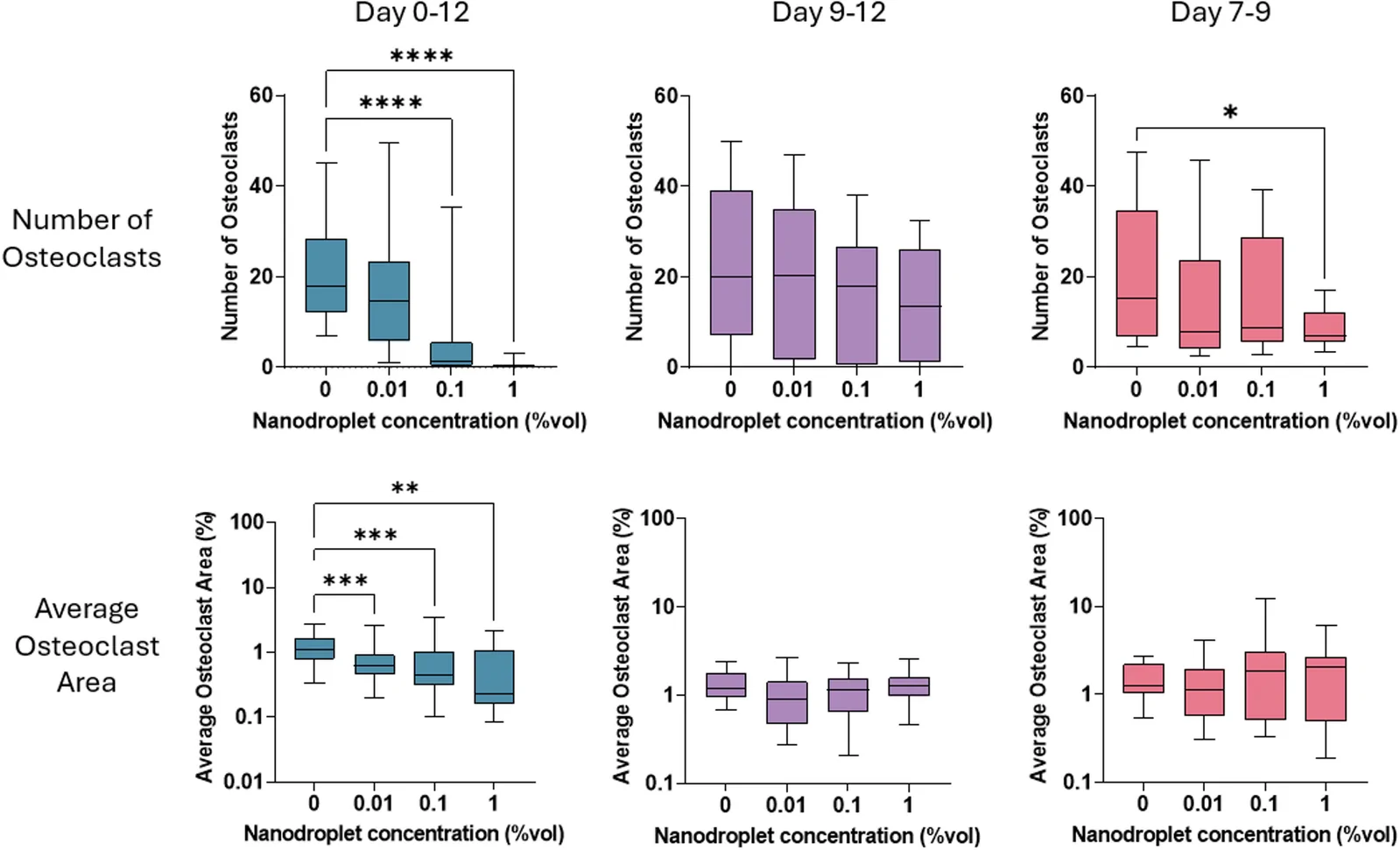
Nanodroplets lead to a dose-dependent reduction in osteoclast number. The top row shows the manual osteoclast count per image for nanodroplet treatment of varying concentrations. The bottom row shows the average osteoclast area in these cultures. The cultures were exposed to nanodroplets for the full 12-day culture (blue), for mature osteoclasts only (purple), or intermittently during fusion (pink). The middle bar represents the median, the box the interquartile range and the error bars the minimum and maximum values. *p < 0.05, **p < 0.01 ***p < 0.001, ****p < 0.0001. *N* = 3, *n* = 6.

This was reflected also on quantification of the data, as shown in Fig. 4, where there was a dose-dependent decrease in osteoclast number when treated with nanodroplets for the full 12-day culture (*p* < 0.0001) as well as when mature osteoclasts only were treated on days 9–12 of culture (although not significant) and when cells undergoing fusion were treated on days 7–9 (*p* = 0.0268). Average osteoclast size was also measured although a difference with respect to nanodroplet treatment was only observed in the full 12-day treatment group. There was a decrease in osteoclast size with increasing nanodroplet concentration (*p* = 0.0007, 0.0003 and 0.0011) and not when added only within day 9–12 and 7–9 windows.

**Fig. 5:**
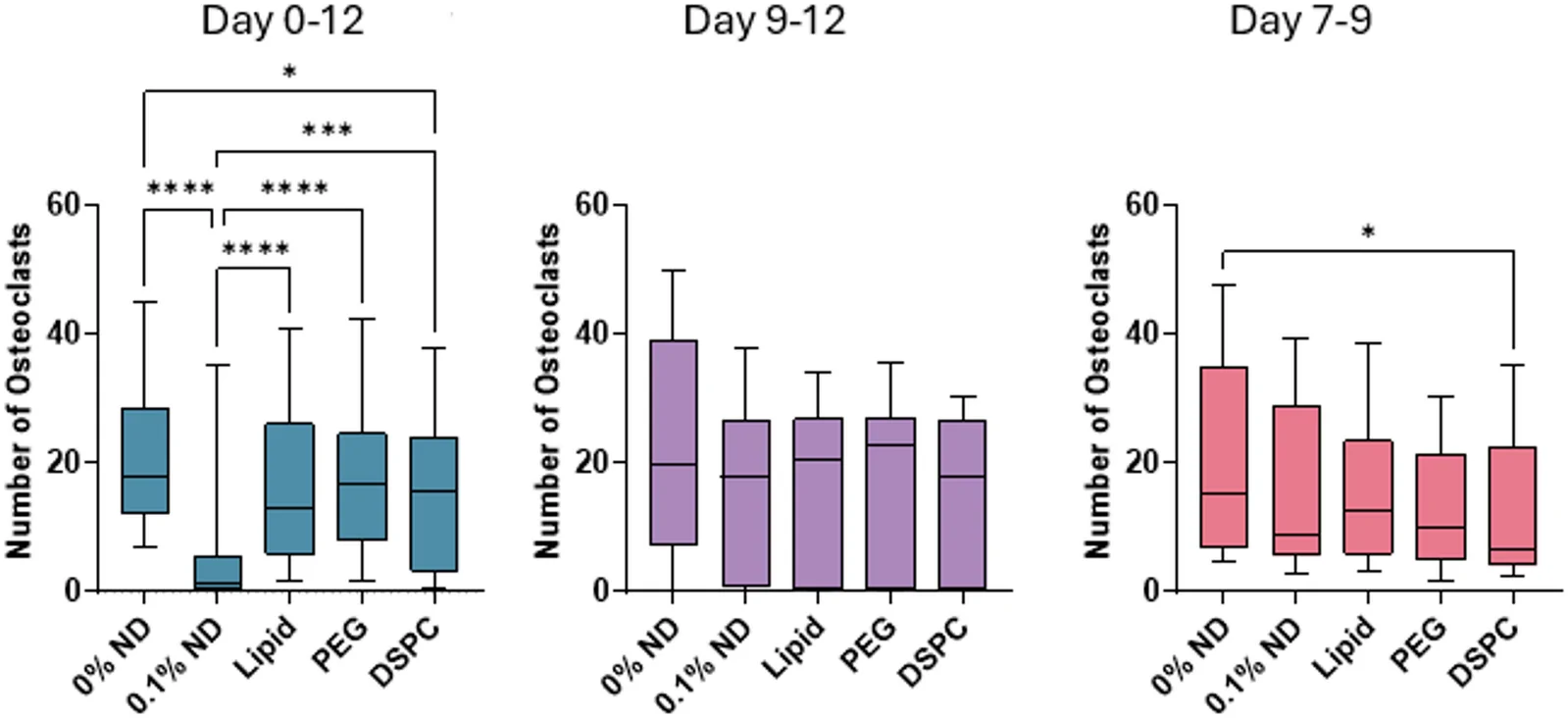
The nanodroplets themselves, not just their lipid shell components, cause a decrease in osteoclast number, especially in the 12-day culture. The manually counted number of osteoclasts after being exposed to nanodroplets for the full 12-day culture (blue), for the presence of mature osteoclasts only (purple) or intermittently during fusion (pink). The middle bar represents the median, the box the interquartile range and the error bars the minimum and maximum values. *p < 0.05, **p < 0.01 ***p < 0.001, ****p < 0.0001. *N* = 3, *n* = 6.

We next hypothesised that the reduction in osteoclastogenesis is due to the lipids that make up the nanodroplet shell rather than the nanodroplets per se. To test this, nanodroplets and their constituents (with the exception of PFP, which is likely to gradually evaporate in cell culture conditions) were incubated with differentiating osteoclasts, and the number of osteoclasts quantified by microscopy. In contrast to our hypothesis, however, there was only a minor decrease in osteoclastogenesis in the case of lipid incubation, while nanodroplet incubation caused a significant inhibition (Fig. 5). Even though the lipid caused a decrease in osteoclast number and size, this effect was found to be amplified by the presence of the PFP core, especially for the full day 0–12 treatment.

**Fig. 6:**
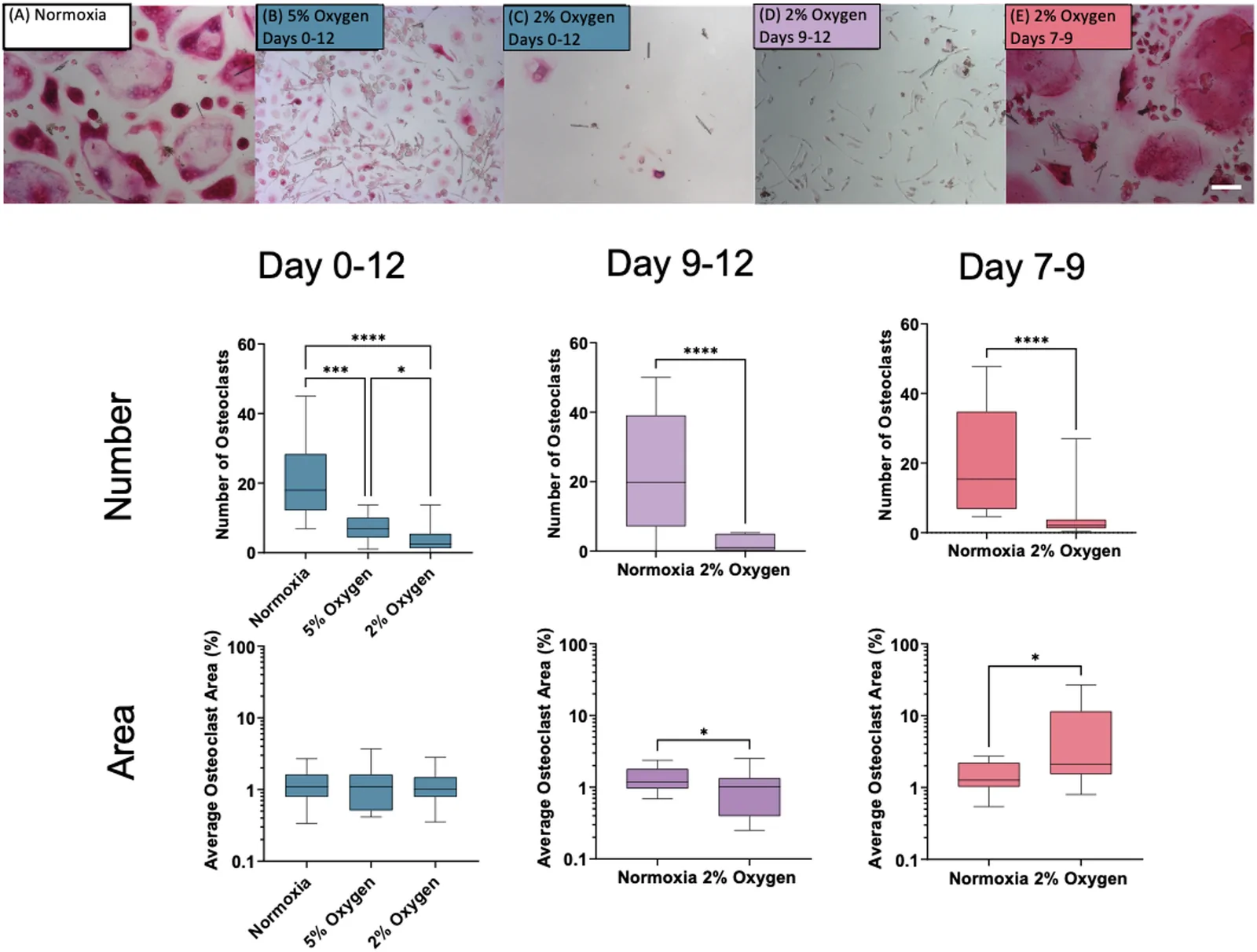
Hypoxia has a schedule dependent effect on osteoclast culture. Representative images are shown for each hypoxic culture condition (A-E). Osteoclast number (top row of graphs) and average osteoclast area per image (bottom row) were acquired by manual counting of osteoclasts. Osteoclasts were exposed to hypoxia for the full 12 days of culture (blue), for mature osteoclasts only (purple) or intermittently during fusion (pink), and were otherwise cultured under normoxic conditions. The middle bar represents the median, the box the interquartile range and the error bars the minimum and maximum values. *p < 0.05, **p < 0.01 ***p < 0.001, ****p < 0.0001. *N* = 3, *n* = 6.

Due to the inert nature of PFP, it was hypothesised that the nanodroplet-specific effect could be due to the high oxygen solubility of PFP, which may increase the available oxygen at the cells. Osteoclasts are very sensitive to oxygen tension with many studies demonstrating effects on activity and osteoclastogenesis^27–29^. Therefore, we explored next whether control over the timing of nanodroplet delivery during osteoclastogenesis could be used to modulate oxygen tension and thus modulate osteoclastogenesis. Prior to testing the effect of nanodroplets directly, we first tested directly the effect of hypoxia on osteoclastogenesis. Osteoclasts were cultured from CD14^+^ PBMCs with three different oxygen schedules: hypoxia for either the full 12-day culture, for mature osteoclasts only (days 9–12) or during late-stage fusion (days 7–9). In all three cases, hypoxia was found to decrease osteoclast number (Fig. 6) but the effect on the osteoclast size was hypoxia schedule dependent. For the full 12-day culture, there was no effect on average osteoclast area. For day 9–12 hypoxic treatment, average osteoclast area significantly decreased. Finally, for day 7–9 hypoxic treatment, average osteoclast area significantly increased. These results indicate that osteoclast fusion is enhanced by a hypoxia-reoxygenation schedule.

**Fig. 7:**
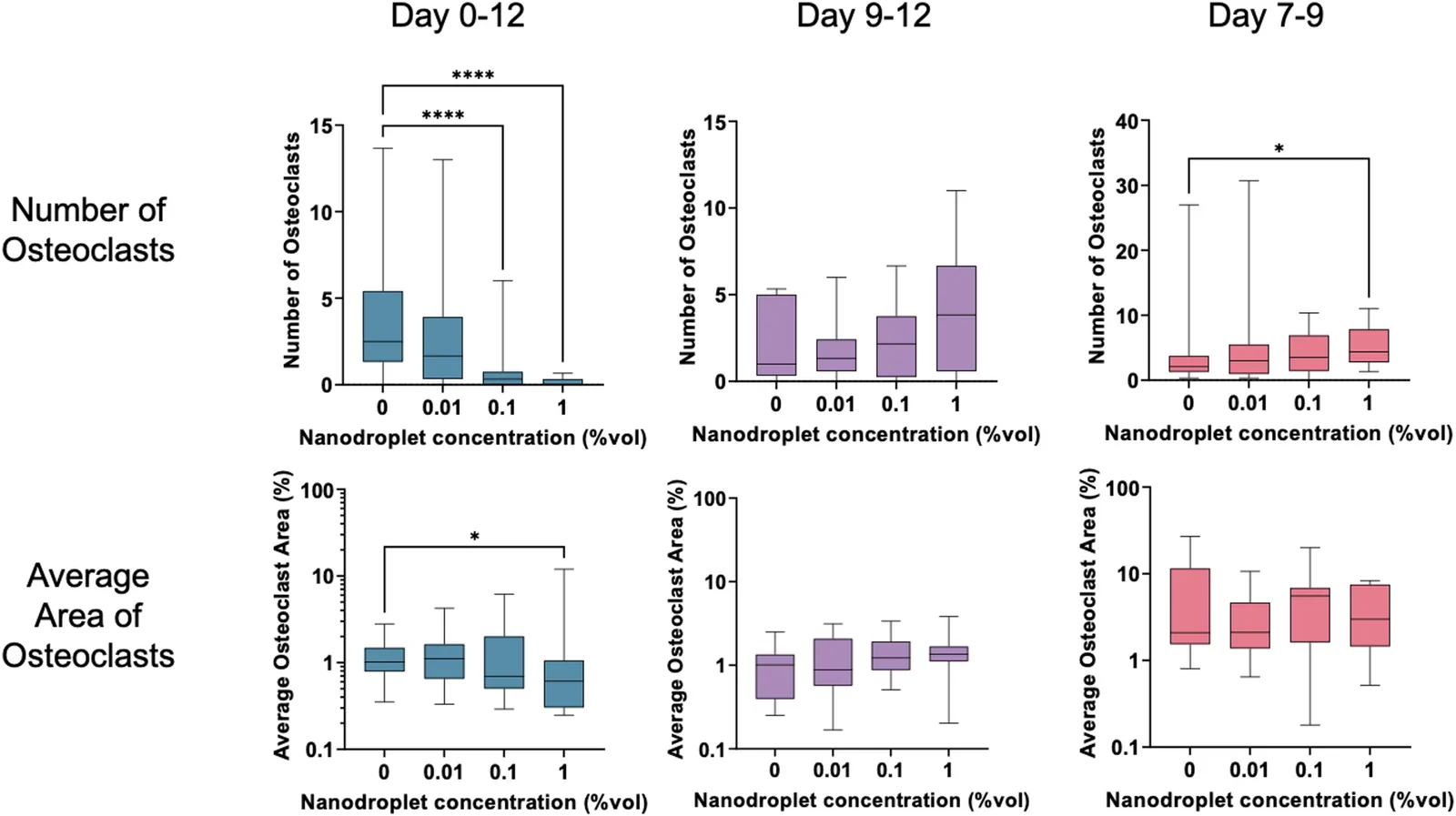
Short term exposure to nanodroplets relieves the effects of hypoxia. Osteoclast number (top row) and average osteoclast area per image (bottom row) were acquired by manual counting of osteoclasts. Osteoclasts were exposed to hypoxia and nanodroplet concurrently for the full 12 days of culture (blue), for mature osteoclasts only (purple) or intermittently during fusion (pink), and were otherwise cultured under normoxic conditions. The middle bar represents the median, the box the interquartile range, and the error bars the minimum and maximum values. *p < 0.05, **p < 0.01 ***p < 0.001, ****p < 0.0001. *N* = 3, *n* = 6.

To determine if nanodroplets could relieve the effects of hypoxia on the osteoclastic cells, we next performed the osteoclastogenesis experiment again in hypoxia and added nanodroplets for the different windows of exposure. For cells treated with nanodroplets in hypoxia, the 12-day exposure still resulted in a decrease in osteoclast number with increasing nanodroplet concentration (Fig. 7. 1%: *p* < 0.0001; 0.01%: *p* < 0.0001). However, for day 7–9 and day 9–12 exposure, the opposite was true, with a visible increase in osteoclast number with increasing nanodroplet concentration including a significant increase in osteoclast number with 1% nanodroplet exposure for days 7–9 (*p* = 0.0198). Overall, nanodroplet treatment partially alleviated the inhibitory effects of hypoxia, although osteoclast numbers did not fully recover to levels observed under normoxic conditions.

## Discussion

This work set out to address the feasibility of the use of nanodroplets in bone repair by investigating the effect of nanodroplets on different bone cell populations. We found that nanodroplets associate with bone cells, especially those derived from monocytes, such as osteoclasts. Furthermore, we found that nanodroplets may alleviate hypoxia-induced effects in osteoclasts in a dose- and schedule-dependent manner. Together, these findings expand current understanding of how nanodroplets operate within bone cells, demonstrating their potential relevance beyond previously investigated use in fields such as tumour treatment.

It is clear from the results that nanodroplets interact with bone cells although localisation assays were not performed and so the specific type or location of association cannot be concluded. Further work seeks to explore this by labelling organelles and assessing co-localisation, for example as in the study by Gratton et al., which used fluorescently labelled nanoparticles and transmission electron microscopy to map intracellular localisation pathways^30^.Although detectable across all bone cell types, interactions were markedly stronger in mononuclear cells in the CD14^+^ PBMC/osteoclast culture (Fig. 1) but did not result in a decrease in cell metabolism (Fig. 2A-D). Cell proliferation in osteoblastic cells exhibited greater complexity, with 0.1% nanodroplets inducing an increase in proliferation in MC3T3-E1 cells and BMSCs (Fig. 2E-G). This is consistent with the work of Polydorou et al. that found microbubbles of the same lipid composition had minimal effect on cells from osteosarcoma cell lines at low concentrations following 24 h of exposure but proliferation was affected at higher concentrations after 24 h of exposure^31^. These microbubbles differ from the nanodroplets in that they consist of a gas core and are larger but the material that is most in contact with the cells, i.e. the lipid shell, has the same composition.

Like osteoblastic cells, CD14^+^ PBMC metabolism was also minimally affected by the presence of the nanodroplets. Whilst osteoclast metabolism was not measured, osteoclast number was significantly affected by the presence of nanodroplets (Fig. 4). For the full 12-day treatment, osteoclast number decreased severely with increasing nanodroplet concentration, but osteoclast size was also found to decrease. This suggests that the nanodroplets could be affecting osteoclast fusion at lower doses, as well as having potential cytotoxic effects at higher concentrations. In fact, the images in Fig. 3 show non-osteoclastic (monocytic) cells still present in the 0.1% and 0.01% v/v treatment groups and the CD14^+^ PBMCs showed no change in metabolism with nanodroplet treatment in Fig. 1D, suggesting that the monocytes present in the culture are not experiencing the same level of cytotoxicity as the osteoclasts. As no direct cytotoxicity assays were performed in the osteoclast culture, future work will be required to disentangle these possibilities and to formally evaluate potential cytotoxic effects of the nanodroplets and how they compete with the effects on osteoclast fusion. However, similar results have previously been observed with nanobubble treatment, where increasing concentration led to a clear decrease in osteoclastogenesis^32^. There are several potential explanations for this result. The first could be that the presence of a foreign entity in the culture stimulates the monocytes to preferentially differentiate into macrophages over osteoclasts. This is supported by the observation in Fig. 1 where the affinity for these mononuclear cells in the CD14^+^ PBMC/osteoclast culture along with the three-dimensional concurrence of the DiO and TRAP staining, indicate enhanced internalisation, possibly by phagocytosis, as has been observed for osteoclasts with uptake of organic and inorganic particles present in prosthetic wear debris^33,34^. Another reason could be that the nanodroplets are preventing the fusion process from taking place. This was the primary explanation given by Knowles et al. for the decrease in osteoclast number with nanobubble treatment, attributing this effect to the lecithin present, which DSPC is a key component of^32^. However, in the present study, the decrease in osteoclast number due to DSPC exposure was not found to be as significant as exposure to the nanodroplets themselves. Finally, another reason could be that the breakdown of the lipid shell following internalisation destabilises the superheated PFP core, causing intracellular vaporisation (a process that results in a significant volume increase), leading to cell death, although this will not be the case for the nanobubbles investigated by Knowles et al.^32^. This last possible explanation is further evidenced in that the lipids that make up the nanodroplets did not have as significant an effect on osteoclast number as nanodroplets, suggesting that the presence of the PFP is integral to the decrease in osteoclast number (Fig. 5). Given the inert nature of PFP^35^, it can be inferred that this significant decrease is not due to PFP cytotoxicity but could be due to a physical disruption of vaporisation of the nanodroplets or the enhanced oxygen solubility capabilities of the nanodroplets.

The nanodroplets’ ability to deliver oxygen was assessed by determining whether they were capable of reversing hypoxia-induced effects. In this study, hypoxic conditions clearly reduced osteoclast numbers, regardless of oxygen concentration, the duration of hypoxia exposure, or the stage of differentiation at which hypoxia was applied. Interpreting these findings in the context of existing literature is challenging, as published reports are often inconsistent and sometimes contradictory. For example, Muzylak et al., Gorrisen et al., and Knowles et al. all found that exposing differentiating osteoclasts to hypoxia led to a decrease in the total number of osteoclasts, consistent with the results in this study^36–38^. Conversely, Arnett et al., Nomura et al., and Murata et al. all found that exposing differentiating osteoclasts to hypoxia led to an increase in the total number of osteoclasts in culture^27,39,40^. However, osteoclast activity and number are not necessarily correlated. Hypoxia has been shown to increase resorption by osteoclasts^27,38,39^. Most notably, Muzalyk et al. found an increase in resorption despite a reduction in cell number. In this case an increase in osteoclast size was also observed, consistent with the results found in the present study when the osteoclasts were exposed to hypoxia for days 7–9^38^. Looking more closely at the size of the osteoclasts formed can provide further insight into whether these changes in cell number are due to increased apoptosis or decreased osteoclast fusion. Larger osteoclasts suggest that fusion is increased and that the decrease in osteoclast number in this study is due to mature osteoclasts experiencing cell death. This conclusion is supported by the work of Arnett et al. who found that mature osteoclast number decreased when exposed to hypoxia^27^. Although, it is worth noting that an increase in osteoclast size could lead to a decrease in osteoclast number due to the number of available monocytes being limited and the number of osteoclasts that can fit in the field of view of the microscope decreasing. Interestingly, altering the timing and duration of the exposure affected how hypoxia influenced osteoclast size. Figure 6 demonstrates that osteoclasts intermittently exposed to hypoxia during the period of most fusion (day 7–9) exhibited increased size, while exposing already mature osteoclasts to hypoxia slightly decreased their size. Conversely, full 12-day hypoxia exposure had no significant effect on cell size. It is notable that all three oxygen conditions led to different outcomes. If it is inferred that osteoclast size correlates with fusion, then the intermittent hypoxic regimen appears to promote fusion where the other two regimens do not. However, the absence of this effect in the 12-day exposure suggests that reoxygenation, rather than hypoxia alone, might be responsible for this change. This is supported by the work of Knowles et al. which showed that reoxygenation can reverse the initial stages of hypoxia-induced cell death in osteoclasts^29^. Reoxygenation has been shown to increase mitochondrial reactive oxygen species^41^ and NF- κb^42^ proteins, both associated with increased osteoclast differentiation^43,44^. It has also been suggested that hypoxia promotes the formation of pre-osteoclasts from monocytes but inhibits subsequent fusion^32,36^. This is supported by the increase in osteoclast area seen with day 7–9 hypoxic exposure, while day 9–12 exposure caused a decrease.

Nanodroplets increased the number of osteoclasts grown under hypoxia over the shorter time frames of days 7–9 and days 9–12 (Fig. 7) compared with untreated controls. This is the opposite effect to that observed in normoxia, where nanodroplet treatment decreased osteoclast number. This suggests that the enhanced oxygen-carrying capacity of the nanodroplets can affect osteoclast viability and/or fusion^32^. However, after 12-days a decrease in osteoclast number and size was still observed, suggesting a long-term cytotoxic effect that outweighs the hypoxia-relief effect. Knowles et al. previously studied oxygen, air and nitrogen loaded nanobubbles for in vitro osteoclast treatment finding that short-term, late stage (day 4–6) osteoclasts in culture had a gas dependent response in terms of osteoclast number and fusion, with further experiments suggesting that this dependence is stimulated by reactive oxygen species rather than HIF-1α^32^. Further study is required to fully understand the mechanism behind the oxygen-dependent nature of the nanodroplets on the osteoclasts such as reactive oxygen species, RANKL and HIF-1α concentration assays as well as resorption assays. It is also important to recognise that osteoclasts play essential roles throughout bone healing, with the number and activity of the cells constantly changing depending on the stage of repair. An initial peak in activity during the inflammatory phase serves to remove necrotic bone and debris, while a second wave during the remodelling phase resorbs redundant callus and reconstructs the lamellar bone^45–47^. In addition to this, osteoclast and osteoblast activity is coupled, with osteoclasts liberating and secreting cytokines that act upon cells of an osteoblastic lineage throughout their differentiation^46,48^. Consequently, any therapeutic strategy involving nanodroplets will need to consider the stage-dependent nature of osteoclast function, ensuring that modulation of osteoclastogenesis aligns with the specific phase of the repair process. This study has begun to attempt to elucidate some of the time dependent effects of the nanodroplets and hypoxia on osteoclasts in vitro by treating them at different stages of their differentiation but for a full understanding of the impact this will have on bone repair, more complex and ultimately in vivo models will be needed.

As an in vitro study, the data provide evidence of direct nanodroplet–cell interactions and context-dependent effects on osteoclastogenesis, but they should be interpreted with caution when extrapolating to the in vivo fracture environment, where vascular perfusion, inflammatory signalling, immune clearance, mechanical loading, tissue architecture and ultrasound activation will all influence nanodroplet behaviour. In addition, although the data suggest that nanodroplets may modulate osteoclast fusion and/or viability, this study did not directly quantify osteoclast apoptosis, resorptive activity, intracellular oxygen tension, HIF signalling or nanodroplet biodistribution after uptake. Future studies should therefore test oxygen-loaded and ultrasound-activated nanodroplets in more physiologically relevant bone repair models, including 3D co-culture or ex vivo bone systems and, ultimately, in vivo fracture models, with direct assessment of oxygen delivery, osteoclast function, biodistribution and effects on bone formation.

## Conclusions

This study investigated the potential of nanodroplets as a carrier that could be used in the future for targeted delivery of bone repair agents. The results indicate that nanodroplets associate with bone cell populations and have minimal cytotoxic effect on osteoblasts or CD14^+^ PBMCs. Additionally, short-term exposure to nanodroplets loaded with oxygen appears to inhibit osteoclast fusion in normoxic conditions but can partially relieve the inhibitory effects of hypoxia on osteoclast survival. In the longer term, however (12 days) exposure to nanodroplets reduced osteoclast number and size. Our in vitro results indicate that PFC nanodroplets alone may have some beneficial effects consistent with net bone formation and warrant further study for the delivery of anabolic agents to enhance bone repair. This study investigated the potential of nanodroplets as a carrier for targeted delivery of bone repair agents. The results indicate that nanodroplets associate with bone cells and have minimal cytotoxic effect on osteoblasts or CD14^+^ PBMCs. Additionally, short-term exposure to nanodroplets loaded with oxygen appears to inhibit osteoclast fusion in normoxic conditions but can partially relieve the inhibitory effects of hypoxia on osteoclasts. In the longer term, however (12 days) exposure to nanodroplets reduced osteoclast number and size.

## Materials and methods

### Nanodroplet fabrication

Nanodroplets were made using the method established by Campbell et al.^49^. DSPC (1,2-distearoyl-sn-glycero-3-phosphatidylcholine, ≥ 99%, HPLC grade) at a concentration of 25 mg/mL in chloroform was purchased from Avanti Lipids. PEG40s was purchased from Sigma-Aldrich and dissolved in chloroform purchased from Fisher Scientific, to achieve a final concentration of 10 mg/mL. The lipids were mixed in a 9:1 molar ratio (DSPC: PEG40S) and left overnight to allow the chloroform to evaporate. To create fluorescent nanodroplets, DiO (Thermofisher) was dissolved in chloroform to form a 1 mg/mL solution and added to the lipid solution to achieve a 90:10:1 (DSPC: PEG: DiO) molar ratio. The lipid mixtures were resuspended in DPBS (Dulbecco’s phosphate buffer solution; Invitrogen) forming a lipid concentration of 5 mg/mL, upon stirring at 500 rpm on a 90 °C hot plate for 45 min, before being sonicated using a Model 120 Sonic Dismembrator with a 3.2 mm diameter tip (Fisher Scientific) for 2.5 min at 40% amplitude setting to achieve a homogenous suspension. 800 µL of the lipid solution was moved to a 1.5 mL Eppendorf tube with 40 µL of PFP (perfluoro-n-pentane; Strem chemicals). The Eppendorf tube was positioned in an ice-water bath, and the sonicator tip was placed 12 mm from the bottom of the Eppendorf tube. The solution was pulse sonicated (2 s on, 15 s off) at 60% amplitude setting for a total sonication time of 90 s.

### Cell line culture

MC3T3-E1 and Saos-2 cells were cultured with high-glucose DMEM supplemented with 10% v/v FBS and 1% v/v 10,000 U/mL penicillin-streptomycin (Lonza, UK). MC3T3E1 cells were seeded at 1.6 × 10^4^ cells/cm^2^ and Saos-2 cells were seeded at 5.2 × 10^4^ cells/cm^2^, 24 h prior to any experiments.

### Bone marrow stromal cell isolation

Primary human bone marrow stromal cells (BMSCs) were isolated from patients undergoing hip replacement surgery at Southampton General Hospital and Spire Southampton Hospital. All isolations were conducted with the written informed consent of the patients and the approval of the local research ethics committee (Local Research Ethics Committee, 194/99). Bone marrow was collected aseptically by surgeons and placed in a universal container. The bone marrow was washed multiple times in plain α-MEM and poured through a 0.6 μm cell strainer to remove fat and bone fragments. The remaining cells were incubated at 37 °C in α-MEM supplemented with 10% v/v FBS and 1% v/v penicillin-streptomycin in a T-175 flask for one week before being washed with PBS and complete media to remove non-adherent cells. Cells were grown to 80% confluency before being trypsinised and seeded at 2.5 × 10^4^ cells/cm^2^.

### Peripheral blood mononuclear cell isolation

CD14^+^ peripheral blood mononuclear cells (PBMCs) were isolated from leukocyte cones obtained from NHS Blood and Transplant (NHSBT) from appropriately consented platelet apheresis donors. The leukocyte cone contents were diluted to 50 mL with a 2 mM EDTA-containing PBS solution before being carefully layered on top of a density gradient separation solution (Lymphoprep, Axis-Shield, UK) in a 2:1 ratio (v/v) across two Falcon tubes. The solution was centrifuged at 800 × g for 20 min at 25 °C with the brake setting turned off. The monocytes were collected from the interphase of the resulting solution and washed four times in cold 2 mM EDTA-PBS solution. The resulting cells were CD14^+^ selected using CD14 human microbeads (Miltenyi, Germany) with magnetic-activated cell sorting (MACS) and seeded at 7.8 × 10⁵ cells/cm² in a 96-well plate.

### Cell-nanodroplet interaction

To test the hypothesis that nanodroplets associate with the cells, fluorescent nanodroplets were made as described above. Mature osteoclasts (Day 10 CD14 + PBMCs cultured in osteoclast media) and BMSCs were treated with 0.1% fluorescent nanodroplets for 48 h. The resulting cultures were then fixed in 4% PFA. Osteoclasts were TRAP stained and the BMSCs were stained using CellTracker Deep Red. Both cell types were then DAPI stained. Imaging was conducted using a Leica TCS-SP8 Laser Scanning confocal microscope (Leica, Germany) using a 40x objective with the following excitation and emission wavelengths: Cell tracker deep red, 630 nm, 655 nm; TRAP, 633 nm, 647 nm; DAPI, 357 nm, 447 nm; DiO, 490 nm, 507 nm. Quantification of the DiO intensity was done using Python. The region of interest was identified using the red stain in each case (osteoblasts: cell tracker deep red; osteoclasts: TRAP) and thresholding. The average pixel intensity was then calculated for the DiO images within and outside the region of interest.

### Viability assays

To determine the effect of nanodroplets on cell metabolic activity and number, Alamar Blue and DAPI cell proliferation assays were performed. For the Alamar Blue assays, the cells were seeded in a 96-well plate with 200 µL of the appropriate media and incubated overnight to adhere. The media was replaced with 90 µL of fresh media and 10 µL of PBS or PBS containing nanodroplets, or their lipid constituents (DSPC and/or PEG(40)s) as a control. After 24 h incubation, the media was again replaced with 100 µL of fresh media containing 10% v/v Alamar Blue and incubated for 2 h for MC3T3E1 and Saos-2 cells and 4 h for BMSCs and CD14^+^ PBMCs. The fluorescence intensity was read using a Glomax plate reader (λ_em_ = 584 nm and λ_ex_ = 520 nm).

For DAPI based cell proliferation assays, cells were seeded in a 24-well plate with 600 µL of complete DMEM. The cells were placed in an incubator for 24 h and then the media was replaced with 540 µL of new media with 60 µL of PBS containing a given volume percentage of nanodroplets. One set of cells was fixed every 24 h for up to 96 h using a 1:1 acetone methanol (Sigma, UK) solution. The cells were then stained using DAPI and imaged using an inverted microscope with a DAPI filter set (λ_em_ = 417–477 nm and λ_ex_ = 357 nm) at 10x magnification. The images were thresholded and the nuclei counted using an ImageJ macro. ( 

### Osteoclast quantification assays

**Fig. 8:**
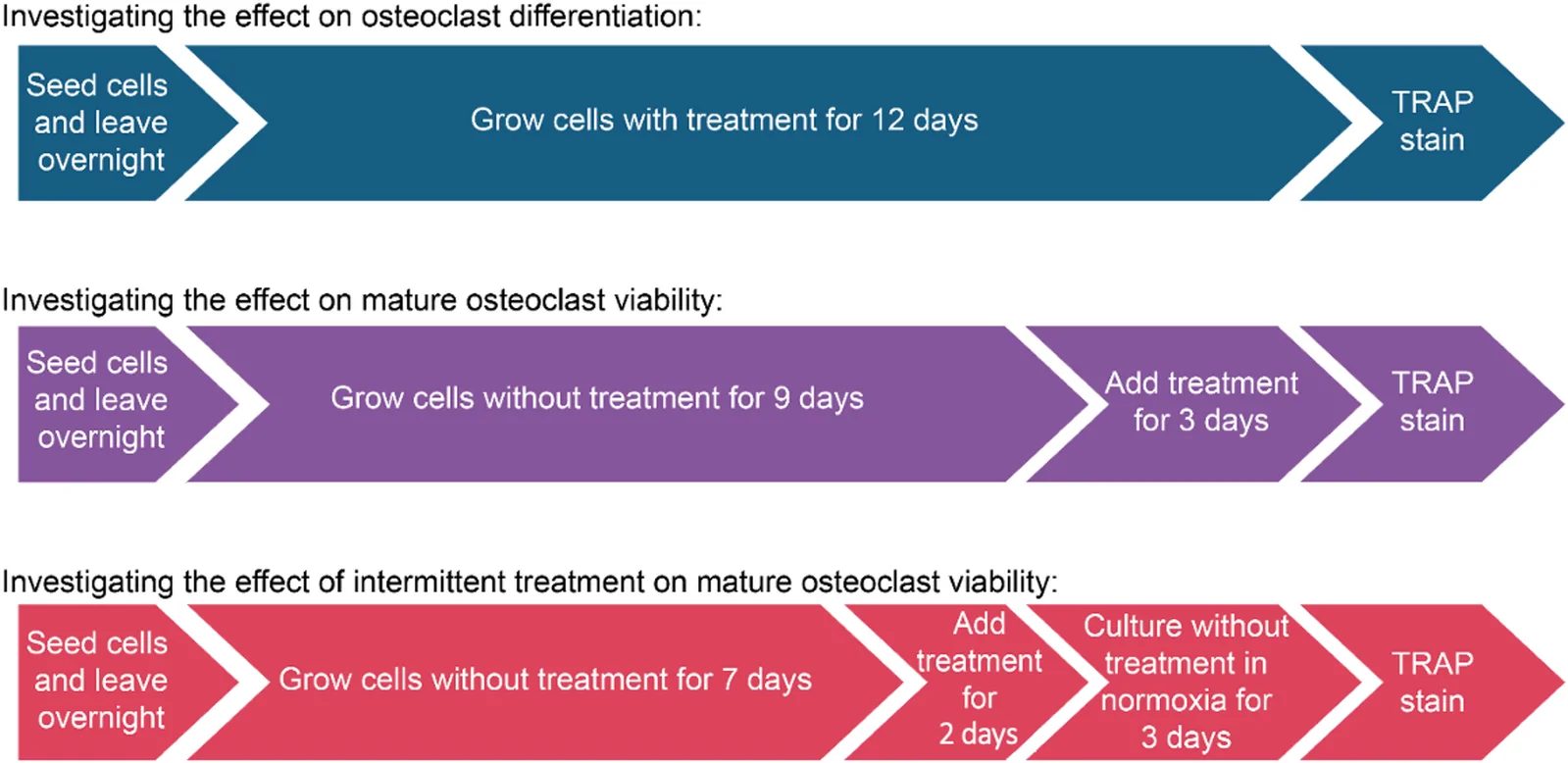
Schematic demonstrating the different treatment regimens for nanodroplet treatment of osteoclast cultures. The experiments were designed to test long term culture with the nanodroplets during fusion, treatment of mature osteoclasts, and short term treatment during fusion.

​​CD14^+^ ​​monocytes were differentiated into osteoclasts in osteoclastogenic media (α-MEM, 10% v/v FBS, 1% penicillin/streptomycin, 1% v/v L-glutamine, 2.7 μM M-CSF, 1.6 μM RANKL) for a total of 12 days. Cells were cultured in seven different groups: three different concentrations of nanodroplets (0.01%, 0.1%, and 1% v/v), as well as 0.1% v/v PEG(40)s, DSPC, a DSPC:PEG(40)s (9:1) lipid mixture, and a no-treatment control. Cells were exposed to the relevant nanodroplet treatment for the entire 12 days, days 9-12, or days 7-9. Where plates were placed in hypoxia (2% or 5% O2), it was for the same duration as the nanodroplet treatment (Figure 8). For the 12-day hypoxic exposure, the plates were intermittently withdrawn from the hypoxic incubator for the media to be changed in a normoxic environment every 3 days. For day 7-9 hypoxic exposure, the media was changed immediately before and after hypoxic incubation and for day 9-12 immediately before, to avoid interrupting the hypoxic environment. When not exposed to nanodroplets and/or hypoxia, the cells were cultured in osteoclastogenic media in a normoxic incubator. The resulting osteoclast cultures were fixed using 4% PFA and washed before undergoing TRAP (tartrate-resistant acid phosphatase) staining. The TRAP staining solutions were prepared according to a protocol from the Center for Musculoskeletal Research, University of Rochester (Center for Musculoskeletal Research, 2020). The stained cultures were then imaged using a Zeiss Axiovert 200 microscope with a 10× objective lens with a colour camera (Zeiss AxioCam color 412-312). The number and average size of osteoclasts per image were quantified by manually outlining each osteoclast in ImageJ.

### Statistical analysis

Statistical analysis was performed using GraphPad Prism version 9.2 software. Where the significance of two groups only was tested, a Mann-Whitney test was performed for unpaired data and a Wilcoxon matched-pairs signed rank test was used for paired data. Where the significance of three or more groups was tested, an ANOVA was performed with multiple comparisons (Tukey for comparing all groups, Dunnett for comparing with control only). For cell association data, fluorescence status (positive or negative) across cell types was compared using a paired Wilcoxon signed-rank test, and a Fisher’s exact test for cytometry data. Statistical significance was defined as *p < 0.05, **p < 0.01, ***p < 0.001, ****p < 0.0001 and non significant (n.s.) as *p* > 0.05.

### Ethics statement

All human tissue was obtained from adults who have provided informed consent and all research was performed in accordance with relevant guidelines/regulations. Primary human bone marrow stromal cells (BMSCs) were isolated from patients undergoing hip replacement surgery at Southampton General Hospital and Spire Southampton Hospital with ethical approval. Leukocyte cones are from the NHS Blood and Transfusion Service Non Clinical Issue (NCI) with ethical approval from the Health Research Authority and has the Research Ethics Committee (REC) reference 18/NW/0231. NHSBT NCI material was supplied anonymised and unlinked to donor identifiers.

## Data Availability

Data will be made available upon reasonable request.
